# Stem Cell Models for Elucidating Cellular Mechanisms of Substance Use Disorders and Advancing Addiction Pharmacology

**DOI:** 10.1155/sci/1717710

**Published:** 2026-08-07

**Authors:** Ji Sun Koo, Huiping Zhang

**Affiliations:** ^1^ Department of Psychiatry, Boston University Chobanian & Avedisian School of Medicine, Boston, Massachusetts, USA; ^2^ Department of Medicine (Biomedical Genetics), Boston University Chobanian & Avedisian School of Medicine, Boston, Massachusetts, USA

**Keywords:** alcohol and drug addiction, embryonic stem cell, induced pluripotent stem cell, microfluidic organoid-on-a-chip, organoid, stem cell models, substance use disorders

## Abstract

Substance use disorders (SUDs) impose major global morbidity and mortality, yet the cellular mechanisms linking genetic risk to neural vulnerability, disrupted neurodevelopment, and drug‐induced neuroadaptations remain poorly understood. Human pluripotent stem cell (hPSC) technologies, including embryonic and induced pluripotent stem cell (ESC/iPSC)‐derived neurons, three‐dimensional (3D) brain organoids, and organoid‐on‐a‐chip platforms, provide scalable, human‐relevant models to address this gap. ESC/iPSC‐derived neuronal cultures enable interrogation of genetic and epigenetic determinants of drug susceptibility and response; cerebral organoids recapitulate tissue architecture and emergent network dynamics; and microfluidic organoid‐on‐a‐chip systems facilitate maturation, enhance reproducibility, and enable controlled exposure paradigms. In this review, we synthesize recent stem cell‐based studies of alcohol, opioid, and stimulant exposure, highlighting insights into neurodevelopmental disruption, synaptic and signaling alterations, neuroinflammation, and network‐level dysfunction. We critically evaluate limitations of stem cell‐based studies, including ethical concerns, inter‐ and intra‐line variability, incomplete cellular maturation, and difficulties in modeling complex circuitry and comorbid conditions. We propose strategies to enhance translational relevance, including standardized differentiation protocols, addition of patient‐derived cells and vascular and immune components, and integration of multi‐omics approaches (transcriptomics, epigenomics, and proteomics) with functional readouts to map molecular pathways underlying drug vulnerability and resilience. Finally, we outline the therapeutic and precision‐medicine potential of stem cell platforms for target discovery, predictive toxicology, and individualized treatment modeling. Despite remaining challenges, stem cell‐based approaches offer a powerful and increasingly tractable path from genetic association to mechanistic insight and therapeutic innovation in addiction research.

## 1. Introduction

Substance use disorders (SUDs), including alcohol and drug addiction, are neuropsychiatric conditions characterized by compulsive substance use despite negative consequences [1]. These disorders affect millions of Americans, resulting in increased mortality, chronic illness, family disruption, and billions of dollars in societal costs each year [2–6]. According to the 2024 National Survey on Drug Use and Health, among individuals aged 12 or older, 58.3% (~168 million people) reported use of tobacco, nicotine, alcohol, or illicit drugs in the past month, and 48.4 million met criteria for SUDs within the past year [7].

Despite advances in neuroscience and psychiatry, effective treatments for SUDs remain limited, and relapse rates are high. The National Institute on Drug Abuse (NIDA) estimates that 40%–60% of individuals in recovery experience relapse [8]. The complexity of neurobiological mechanisms underlying addictive behaviors poses significant challenges for research. Traditional models, including immortalized cell lines, animal models, and postmortem human brain tissue, have provided valuable insights into the molecular processes of addiction [9, 10]. Nevertheless, these models have limitations, often failing to recapitulate the intricate neural circuits and systemic interactions critical for human therapeutic development [11].

Recent advances in stem cell biology offer new opportunities to address these challenges. Human pluripotent stem cells (hPSCs), such as embryonic stem cells (ESCs) and induced pluripotent stem cells (iPSCs), can be differentiated into diverse neural and glial subtypes implicated in the addiction pathophysiology [12]. Unlike traditional models, stem cell‐derived systems allow for the investigation of human‐specific genetic contributions, cellular diversity, and the complex neural circuitry involved in addiction [13]. In addition, three‐dimensional (3D) brain organoids and engineered organoid‐on‐a‐chip platforms create physiologically relevant microenvironments that facilitate studies of cellular interactions, neural networks, and drug responses in vitro [14–16].

In this review, we summarize the current landscape of stem cell‐based models for studying alcohol and drug addiction, highlighting their advantages over traditional cell and animal models. We discuss recent breakthroughs in modeling addiction, explore strategies to overcome existing limitations, and evaluate the translational potential of these systems for effective therapeutic interventions.

## 2. Literature Search

A systematic literature search was conducted using the search terms “stem cell” AND ("addiction” OR “substance use disorders") across PubMed, Google Scholar, and Medline databases, with searches spanning from September 2025 through March 24, 2026. Only the peer‐reviewed articles published in English were included. Titles, abstracts, and methods sections were screened for relevance, and full texts were obtained for articles meeting the inclusion criteria. The review focused on studies addressing alcohol and drug addiction; studies on other psychiatric disorders were excluded. The initial search identified 372 articles. Of these, 186 met inclusion criteria (focused on alcohol and drug addiction), while 186 were excluded for addressing other psychiatric disorders. Among the included studies, 35 examined ESC or iPSC‐derived models, 36 investigated organoid models, and 30 explored organoid‐on‐a‐chip systems applied to alcohol and drug addiction research. The remaining 85 articles described conventional models, including cell lines, animal studies, and human postmortem brain studies focused on alcohol and drug addiction, as well as stem cell studies related to other psychiatric disorders. The following sections examine traditional experimental approaches alongside stem cell‐derived models in addiction research.

## 3. Conventional Experimental Models for Addiction Research: Benefits and Challenges

### 3.1. The Cell Line Model

Cell lines have traditionally been widely utilized in neuroscience research due to their reproducibility, ease of culture, and cost effectiveness [17]. These cell lines are employed across diverse research fields, including studies of gene function, antibody production, drug metabolism, and vaccine development [18–20]. By using cell cultures, researchers can investigate protein functions and molecular mechanisms underlying disease development, thereby advancing the understanding of disease pathophysiology [21]. In neuroscience, commonly used cell culture models include neuronal cultures derived from rats and mice. These models enable the examination of species‐specific differences in metabolic processes, disease phenotypes, and pathogenesis relative to humans [22]. However, significant differences in gene expression and transcription factor signaling between rodents and humans highlight the limitations of rodent systems and necessitate the careful evaluation of conserved molecular pathways [23, 24].

The SH‐SY5Y cell line, derived from human neuroblastoma, can differentiate into neuron‐like cells exhibiting dopaminergic characteristics, making it especially relevant for alcohol and drug addiction research [25]. Furthermore, studies demonstrate that different differentiation protocols preferentially generate specific neuronal subtypes, including adrenergic, cholinergic, and dopaminergic neurons [26, 27]. Fully differentiated SH‐SY5Y cells express a broad range of markers indicative of mature neurons, including structural proteins (βIII‐tubulin [Tuj1], microtubule‐associated protein 2 [MAP2], neurofilament proteins [NF‐L, NF‐M, NF‐H], and tau), synaptic and vesicle‐associated proteins (synaptophysin [SYN], synaptic vesicle protein 2 [SV2], synapsin I, and postsynaptic density protein 95 [PSD‐95]), and neurotransmitter‐related proteins (tyrosine hydroxylase [TH], choline acetyltransferase [ChAT], dopamine transporter [DAT], and vesicular glutamate transporter [VGLUT]) [28–30]. They also express neuron‐specific enolase (NSE), growth‐associated protein 43 (GAP‐43), and neuronal differentiation‐associated transcription factors, such as NeuroD1 and BRN [21]. Importantly, being human‐derived, SH‐SY5Y cells express several human‐specific proteins and isoforms absent in primary rodent cultures, providing a unique platform for modeling human neuronal biology [28]. SH‐SY5Y cells have been utilized to investigate various neurological diseases such as Parkinson’s disease, Alzheimer’s disease, depression, and schizophrenia [31–34]. Due to their dopaminergic properties, SH‐SY5Y cells are particularly valuable for addiction research and have been utilized to study cocaine, methamphetamine, nicotine, and alcohol use disorders (AUDs) [35–39].

While cell culture models such as SH‐SY5Y provide valuable mechanistic insights, they have notable limitations: (1) immortalization can alter key signaling pathways and generate artificial responses divergent from native physiology [40–42] and (2) they lack the tissue architecture, glial contributions, and complex synaptic interactions essential for modeling circuit plasticity underlying addiction [43–46]. Despite their utility as accessible and cost‐effective tools for preliminary studies, these limitations underscore the need for more sophisticated, human‐specific models to fully capture the pathophysiology of alcohol and drug addiction.

### 3.2. The Animal Model

Animal models have been essential for advancing our understanding of addiction, particularly in elucidating reward circuitry and identifying molecular pathways responsive to drug exposure [47]. These models have been extensively used across nearly every major class of addictive substances, including stimulants such as cocaine and amphetamines, opioids, alcohol, nicotine, and cannabinoids [48–52]. In addiction research, drug self‐administration (SA) paradigms are commonly employed, where drug delivery depends on the animal’s motivation to consume the substance [53]. Both the duration of drug exposure and the temporal pattern of delivery were manipulated to model addiction‐relevant behaviors.

Nevertheless, fundamental species differences limit the translational relevance of these findings [54]. Rodent and human brains differ significantly in structure and function, especially regarding prefrontal cortex organization, reward circuitry, and complex cognitive processes [55, 56]. Notably, the density, distribution, and function of key neurotransmitters such as dopamine, glutamate, and GABA vary considerably between rodent and human brains [57–59]. These differences suggest that neurotransmitter‐mediated drug responses observed in rodents may not fully replicate human neurobiological dynamics, potentially limiting translational applicability.

Moreover, while animal models can effectively simulate drug SA, addiction, and withdrawal, they fail to capture complex human‐specific factors such as social and environmental influences, craving, decision‐making processes, and psychiatric comorbidities [60–62]. Differences in drug metabolism between species further complicate translation. Humans and rodents express varying numbers and types of drug‐metabolizing enzymes, particularly within the cytochrome P450 (CYP) family, with humans possessing approximately eight CYP genes compared to about 34 in mice [63, 64]. These metabolic discrepancies, alongside neurodevelopmental and cognitive differences, contribute to divergent drug responses and behaviors related to craving, relapse, and compulsive drug seeking, challenging the accurate modeling of these key addiction features. Consequently, many pharmacological treatments that reduce drug‐seeking behaviors in animals fail to translate effectively to humans, emphasizing the need for complementary human‐specific models to better understand and target the addiction [65, 66].

### 3.3. Human Postmortem Brain Study

Human postmortem brain tissue studies have provided critical insights into cellular, molecular, and structural alterations associated with chronic substance use. These studies enable direct examination of affected human brain tissue, allowing analyses of gene and protein expression changes and other biochemical alterations linked to alcohol and drug addiction [67, 68]. For example, transcriptomic analyses of human AUD brains have uncovered key miRNA‐mRNA regulatory networks implicated in neuroplasticity and neuroinflammation [69].

However, postmortem tissues represent a static snapshot that cannot capture dynamic cellular processes underlying addiction onset, progression, and persistence. The postmortem interval (PMI) introduces challenges as brain tissue undergoes degradation shortly after death, with genetic material, proteins, and other biomolecules rapidly deteriorating, thereby impacting the reliability of molecular analyses [70, 71]. Experimental modeling of PMI in mice demonstrated a significant loss of disease‐specific molecular signatures with an increasing delay before tissue preservation [72]. Similar degradative processes likely occur in human brains, potentially obscuring biomarker identification for neuropsychiatric disorders including alcohol and drug addiction [73, 74].

Additionally, postmortem studies face confounding variables including polysubstance use, psychiatric comorbidities, medication history, and cause of death heterogeneity [75–77]. For instance, a review of medical examiner toxicology reports from 130 postmortem schizophrenia cases (National Institute of Mental Health, NIMH) revealed that while 88% of subjects underwent toxicology screening, 13.8% tested positive for antipsychotic medications [76, 78]. Accounting for such factors is crucial as medication exposure can alter neurochemistry, gene expression, and cellular structure, thereby confounding disease‐specific interpretations.

In summary, although postmortem brain studies provide valuable information about the neurobiology of alcohol and drug addiction, they are inherently limited in capturing the dynamic, causal, and temporal mechanisms driving the addiction initiation and progression.

Taken together, traditional cellular, animal, and postmortem models have provided important insights but are limited by species‐specific differences and the inability to capture dynamic, patient‐specific responses. Table 1 summarizes the key limitations of commonly used animal and in vitro models, underscoring the need for human stem cell‐based systems.

**Table 1:** Conventional experimental models for alcohol and drug addiction research.

Model type	Utilization	Pros	Cons	References
Cell lines derived from animals	Used to study protein function, drug metabolism, gene expression, and more	Reproducibility, ease of culture, low cost	Significant differences between species	Majumdar et al. [20]; Gambardella et al. [18]; Jin et al. [19]; Kaur & Dufour [17]; Wong et al. [24]
Cell lines derived from humans	Used to study cellular mechanisms, drug metabolism, gene modifications, and more	Human cell lines (e.g., SH‐SY5Y) have neuron‐like characteristics; used to model human neuronal biology	Cannot capture functional diversity of neuronal and glial cells; Lack tissue architecture and complex synaptic interactions; Cell immortalization alters key signaling pathways.	Kovalevich & Langford [28]; Lopez‐Suarez et al. [25]; Kulatunga et al. [29]; Damuka et al. [35]; De Bardet et al. [40]
Animals such as mice and rats	Used to examine reward circuity and molecular pathways underlying addiction	Model addiction through self‐administration	Differences between rodents and humans in complex cognitive processes and reward circuit pathways	Kim and Im [50]; Zuo et al. [52]; Kuhn et al. [47]; Venniro and Shaham [53]; Wildenberg et al. [59]; Le Bras [64]
Human post‐mortem brain tissues	Used to examine brain structural and molecular changes‐associated with addiction	Analysis of gene/protein expression changes and other alterations induced by substance use	Cannot capture dynamic processes underlying addiction; Degradation may occur hours after death	Cabrera‐Mendoza et al. [67]; Javan et al. [68]; Thakral et al. [71]; Olney et al. [72]; Mallory et al. [73]

## 4. Stem Cell‐Derived Models for Addiction Research: Benefits and Challenges

Stem cell‐derived models overcome many limitations of traditional approaches (Table 2). The following sections detail specific stem cell platforms (ESCs, iPSCs, organoids, and organoids‐on‐chips) and their applications in addiction research.

**Table 2:** Stem cell‐derived models for alcohol and drug addiction research.

Model type	Utilization	Pros	Cons	References
ESCs: Derived from the inner cell mass of blastocytes after deconstruction of the embryo	Platform to study drug‐induced neurotoxicity, synaptic remodeling, and gene expression changes	Potential to generate variety of brain cells; ability to model specific brain regions implicated in addiction	Ethical concerns	Damdimopoulou et al. [79]; Varzideh et al. [80]; Hosseini et al. [81]; Jamalpoor et al. [82]; Deehan et al. [83]; Lossi et al. [84]
iPSCs: Derived from adult somatic cells that are programed into pluripotent state	Used to study cellular responses, complex neural circuits, and brain region‐specific responses to substances; can be applied to personalized therapeutic testing and drug screening	Can differentiate into different neuronal and glial subtypes; patient‐specific and preserve the genetic and epigenetic background of patients	Challenges include variability in reprograming efficiency, incomplete neuronal maturation, and the need for optimized differentiation protocols	Oh & Jang [85]; Desprat et al. [86]; Hong and Do [87]; Paik et al. [88]; DeBoever et al. [89]; Madrid et al. [90]; Kirkeby et al. [91]
Organoids: Derived from ESCs or iPSCs; self‐organizing 3D structures	Used as patient‐specific systems to model genetic risk factors, epigenetic modifications, and individual variability in response to substances; region‐specific models like the midbrain or cortical organoids; can be used to study addiction‐related circuits	3D structures exhibiting key characteristics of human brain physiology; gene expression profiles of organoids resemble that of human fetal brain; contains a diverse population of neuronal and glial cells	Challenges include growth variability, lack of full vascularization, and difficulties in achieving mature neuronal phenotypes	Miura et al. [92]; Mancinelli et al. [93]; Wang et al. [94]; Ajongbolo and Langhans [95]; Aquilino et al. [96]
Organoids‐on‐chips: Integration of brain organoids with microfluidic devices; allows for precise control of fluid flow, nutrient delivery, and chemical gradients	Used to model substance exposure and simulate fluctuations in drug concentration and time of use; able to incorporate multiple organoids representing different brain regions to model circuits underlying addiction	Can recreate functional units of organs and recapitulate human disease with incorporation of biochemical signals; the precision of organoid microfluid technology mimics the brain’s dynamic microenvironment such as circulation with integration of vascular‐like channels	Challenges include technical complexity, high cost, and standardization needs	Zhou et al. [97]; Adlakha [98]; Zhang et al. [99]; Kshirsagar et al. [100]; Pagliaro et al. [101]; Kistemaker et al. [100]; Rizzuti et al. [102]

### 4.1. Human ESCs

**Figure 1:**
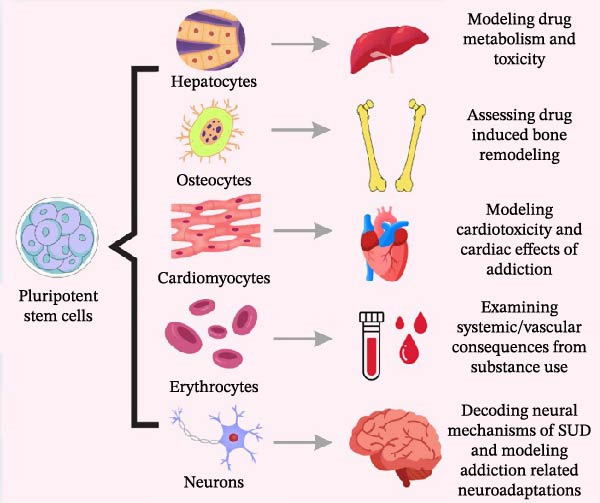
Stem cell differentiation into specialized cell types for disease modeling. Human pluripotent stem cells (hPSCs) can be differentiated into diverse cell types including hepatocytes, osteocytes, cardiomyocytes, erythrocytes, and neurons. These differentiated models enable investigation of molecular and cellular mechanisms underlying substance use disorders (SUDs) and other diseases.

Human ESCs are derived from the inner cell mass of blastocysts, requiring embryo disassembly [79]. ESC‐derived cells are genetically human and offer more accurate disease modeling compared to traditional systems. As illustrated in Figure 1, ESCs exhibit unlimited self‐renewal and pluripotency, the ability to differentiate into all cell types of the body [79, 80]. In neuroscience and psychiatry, ESCs can generate various brain cell types, including dopaminergic, GABAergic, and glutamatergic neurons, as well as astrocytes, oligodendrocytes, and microglia [81]. This broad differentiation potential allows modeling of specific brain regions implicated in addiction, such as the prefrontal cortex, nucleus accumbens, and the ventral tegmental area. It also facilitates the study of cell‐specific mechanisms, pathways, and interactions underlying alcohol and drug addiction by observing neuronal subtype responses to addictive substances in affected versus unaffected individuals.

ESCs provide an early developmental baseline free of social and environmental influences, enabling the exploration of how addictive substances alter neural differentiation and circuit assembly. ESC‐derived neurons exposed to substances allow investigations into drug‐induced neurotoxicity, synaptic remodeling, and gene expression changes [82–84]. However, ethical concerns and regulatory constraints surrounding ESC use have accelerated the adoption of alternative models such as iPSCs [103].

### 4.2. iPSCs

Human iPSCs are generated by reprograming adult somatic cells to a pluripotent state through expression of key transcription factors [85]. Phenotypically, morphologically, and antigenically, iPSCs closely resemble ESCs [104]. Similar to ESCs, iPSCs can differentiate into diverse neuronal and glial subtypes, enabling the study of complex neural circuits and brain region‐specific processes involved in the addiction [87]. Unlike ESCs, iPSCs are derived from accessible somatic tissues such as skin, blood, dental tissue, or urine [86], allowing the modeling of individual susceptibility factors including inherited genetic risk, gene–environment interactions, and prior substance exposure.

iPSCs retain the genetic and epigenetic background of the donor, making them ideal for investigating patient‐specific responses [88, 89]. iPSC‐derived neurons and glia facilitate studies of cellular responses to substances, synaptic connectivity alterations, neurotransmitter signaling, and gene expression changes [105, 106]. Additionally, iPSC systems enable drug screening and personalized therapeutic testing, bridging the molecular discovery to clinical intervention [90, 91]. Despite ethical advantages over ESCs, the iPSC technology faces challenges including variability in reprograming efficiency, incomplete neuronal maturation, and the need for optimized differentiation protocols.

### 4.3. Brain Organoids

Brain organoids are 3D self‐organizing structures derived from ESCs or iPSCs that recapitulate key aspects of human brain physiology [107, 108]. Their gene expression profiles resemble those of the human fetal brain, providing innovative models for neuroscience and psychiatric research [109]. Under specific culture conditions, pluripotent stem cells differentiate into diverse neuronal and glial populations arranged in layered structures like the developing cortex [109]. This structural complexity enables the study of cell–cell interactions, synaptic connectivity, and network activity in a more physiologically relevant microenvironment than traditional models.

**Figure 2:**
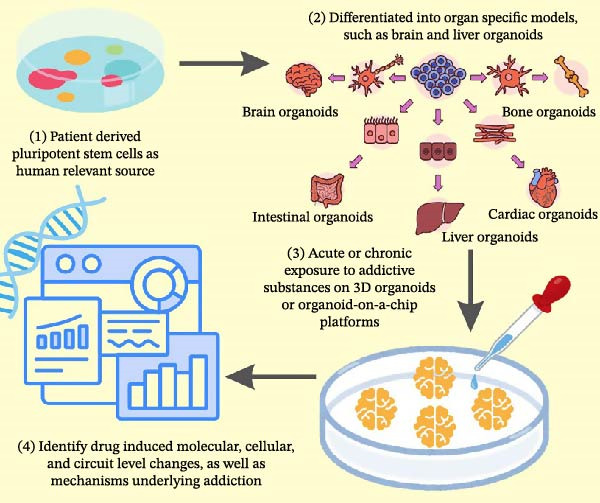
Patient‐derived iPSC organoid models for mechanistic investigation of addiction. Induced pluripotent stem cells (iPSCs) are generated by reprograming somatic cells obtained from patients. Patient‐derived iPSCs are differentiated into organoids representing brain, bone, cardiac, intestinal, and liver tissues. These organoids are exposed to addictive substances in either acute or chronic timeframes. Molecular, cellular, and circuit‐level changes induced by drug exposure are investigated in vitro to elucidate mechanisms of substance use disorders and potential therapeutic targets.

As shown in Figure 2, brain organoids facilitate examination of how addictive substances influence neurodevelopmental trajectories including synapse formation, neuronal maturation, and circuit assembly [92, 93, 110, 111]. Organoids allow the investigation of drug‐induced toxicity and plasticity at cellular and network levels [112–114]. Patient‐specific iPSC‐derived organoids can model genetic risk factors, epigenetic modifications, and individual variability in substance responses [94–96]. Advances in organoid techniques have enabled regional brain patterning, including midbrain or cortical organoids, facilitating studies of addiction‐related circuits [115]. These models have been employed to investigate the mesolimbic dopamine “reward” pathway, whose alterations are linked to alcohol and drug addiction and other neuropsychiatric disorders [116, 117]. Despite their utility, organoids face challenges such as growth variability, lack of full vascularization, and difficulties in achieving mature neuronal phenotypes [118–120]. Nevertheless, they represent a physiologically relevant platform bridging cellular mechanisms with circuit‐level phenomena in addiction research.

### 4.4. Organoids‐on‐Chips

Organoid‐on‐a‐chip platforms integrate stem cell‐derived brain organoids with microfluidic devices that precisely control fluid flow, nutrient delivery, and chemical gradients [121]. These systems recreate functional organ units and replicate homeostasis and disease processes by incorporating biochemical and biomechanical cues [122–125]. This technology enhances physiological relevance by mimicking the brain’s dynamic microenvironment, including circulation, metabolite exchange, and signaling molecule exposure [97, 126].

Organoid‐on‐chip platforms enable controlled, reproducible substance exposure paradigms that mimic human‐like fluctuations in drug concentration and timing [127]. Advances also permit integration of multiple organoids representing different brain regions, facilitating modeling of circuit‐level communication among areas central to addiction, such as the cortex, striatum, and midbrain [98, 99, 101, 128, 129]. The microfluidic environment supports the incorporation of vasculature‐like channels and immune components, enhancing investigations into blood‐brain barrier interactions and neuroinflammation triggered by addictive substances [100, 102, 130]. High experimental control also enables comprehensive pharmacological profiling, bridging molecular discovery and preclinical evaluation [131, 132]. Although challenges remain, such as technical complexity, high cost, and the need for standardization, organoid‐on‐chip platforms represent a promising next‐generation tool for dissecting the addiction mechanisms and evaluating targeted therapies [133, 134].

## 5. Stem Cell‐Derived Cellular Models for Studying Opioid, Alcohol, and Stimulant Use Disorders

Recent advances in stem cell research have positioned ESCs and iPSCs as valuable models for investigating the neural mechanisms underlying alcohol and drug addiction (Table 3). These cells are also employed in drug discovery, toxicity testing, and exploring potential therapeutic interventions for alcohol and drug addiction. By differentiating into diverse neuronal and glial subtypes, these models enable the investigation of how addictive substances affect the human brain at cellular, molecular, and circuit levels [144–146]. Moreover, iPSC models preserve the genetic and epigenetic backgrounds of individual patients, allowing researchers to study patient‐specific risk factors and variability in drug responses [147, 148]. These advantages overcome the limitations of traditional animal models, such as species differences, thereby filling a critical gap in addiction research.

**Table 3:** Stem cell‐derived cellular models for alcohol and drug addiction research.

Model type	Tissues origins/cell types	Types of substances	Findings	References
iPSC	Neurons derived from individuals with OUD and CUD	Opioid use disorder (OUD); cocaine use disorder (CUD)	Morphine and cocaine treated neurons showed gene expression changes overlapping with OUD/CUD brain tissue; EGR1 dysregulated in both models	Mendez et al. [135]
iPSC	Dopaminergic neurons derived from OUD and control subjects	Opioid use disorder (OUD)	Valproic acid altered the expression of dopaminergic regulators DAT, Nurr1, TH, and Drd2, especially in opioid dependent lines	Sheng et al. [136]
iPSC	Neural midbrain progenitors exposed to morphine and withdrawal conditions	OUD (prenatal/developmental)	Extended neural induction increased opioid receptor expression; withdrawal disrupted cAMP signaling and shifted cell fate decisions	Sullivan et al. [137]
iPSC	Brainstem preBötzinger complex (preBötC) like neurons critical for respiratory rhythm	OUD (respiratory depression)	Modeled opioid induced respiratory depression and recovery; exposure to opioids (DAMGO, fentanyl, methadone, codeine) induced dose‐dependent inhibition of neuronal firing; identified IC_50_ values comparable with literature	Guo et al. [138]
hESCs and iPSCs	Embryonic and induced pluripotent stem cells differentiated into neural and placental lineages	Alcohol use disorder (AUD); prenatal alcohol exposure	Alcohol exposure altered DNA methylation and DPPA4 expression in both tissues; exposure also disrupted early differentiation and neurodevelopmental gene networks	Auvinen et al. [139]
hESCs and iPSCs	hPSC derived cortical neurons	AUD (fetal alcohol spectrum disorders)	Chronic alcohol exposure disrupted patterning and synaptic function; upregulation of WNT led to a shift in regional identity: caudal forebrain markers increased while anterior markers decreased	Fischer et al., [140]
iPSC	Microglia from AUD individuals	AUD	Microglia from high AUD polygenic risk score individuals showed increased phagocytosis, CLEC7A expression, and synaptic pruning after ethanol exposure	Li et al. [127]
iPSC	Human cardiomyocytes derived from iPSCs	AUD (cardiotoxicity)	Chronic ethanol caused oxidative stress, abnormal calcium handling, apoptosis, and contractile dysfunction; exposure altered WNT and TGFβ pathways.	Liu et al. [141]
iPSC	Human cardiomyocytes	Stimulant use disorder (cocaine, amphetamines, MDMA, NPS)	Modeled cardiotoxic effects of stimulant use; exposure to stimulants altered beat rate, amplitude, and repolarization in a concentration‐dependent manner	Zwartsen et al. [142]
iPSC	Human neocortical neurons	Stimulant use disorder (cocaine)	Cocaine accelerated neuronal differentiation and neurogenesis. Effects mediated by CYP450 metabolism	Kindberg et al. [143]

This section reviews emerging studies utilizing ESC‐ and iPSC‐derived cellular models to investigate opioid, alcohol, and stimulant use disorders. These platforms have revealed drug‐induced alterations in neuronal physiology, gene expression, and glial function and identified novel therapeutic targets.

### 5.1. ESC‐ and iPSC‐Derived Cellular Models for Studying Opioid Use Disorder (OUD)

Patient‐derived iPSCs have proven particularly valuable for understanding how genetic risk factors shape cellular responses to addictive substances, offering mechanistic insights into individual variability in addiction susceptibility. A compelling example comes from an iPSC study that compared gene expression profiles between morphine‐ and cocaine‐treated neurons derived from iPSCs and postmortem brains of individuals with OUD and cocaine use disorder (CUD), revealing convergent patterns [135]. Morphine treatment induced gene expression changes in neurons derived from an individual who died of opioid overdose, mirroring those observed in the OUD brain tissue. Notably, both showed differential expression of the immediate early gene EGR1, known to be dysregulated by opioid use [135]. Another study used iPSC‐derived dopaminergic neurons from opioid‐dependent and control subjects to examine the effects of a DAT (SLC6A3) genetic variant [136]. This work provided the first evidence that the 3^′^ variable number tandem repeat (VNTR) polymorphism in the human DAT gene influences DAT expression in human dopaminergic neurons. Furthermore, valproic acid exposure altered the expression of dopaminergic regulators, including DAT, Nurr1, TH, and Drd2, with the most pronounced effects in opioid‐dependent lines. These findings highlight the interplay between genetic variation and environmental factors in shaping dopaminergic neuron function, providing a human‐relevant model to study the mechanisms underlying OUD.

Building on these transcriptomic parallels, a recent work used iPSC‐derived midbrain progenitors to model prenatal morphine exposure and withdrawal [137]. Sullivan et al. demonstrated that extended neural induction enhances the opioid receptor expression, enabling more physiologically relevant studies of opioid signaling. Morphine withdrawal disrupted cAMP signaling and altered neural fate decisions, maintaining progenitor status during chronic exposure but promoting neuronal differentiation at the expense of astrocyte formation during withdrawal [137]. Complementing these studies, recent efforts generated iPSC‐derived preBötzinger complex (preBötC)‐like neurons, critical for respiratory rhythm, to study opioid‐induced respiratory depression. This model facilitates mechanistic investigations of opioid overdose and recovery and provides a platform to test therapies for life‐threatening respiratory effects [138]. Collectively, these iPSC‐based approaches capture the genetic, molecular, developmental, and physiological consequences of opioid exposure, offering versatile human‐relevant platforms to study OUD and related complications.

### 5.2. ESC‐ and iPSC‐Derived Cellular Models for Studying AUD

Human ESCs and iPSCs are powerful tools for studying AUD and prenatal alcohol exposure (PAE) in physiologically relevant systems. By differentiating these cells into neural progenitors, dopaminergic neurons, cortical neurons, microglia, cardiomyocytes, or organoids, researchers can explore how alcohol affects gene expression, epigenetic regulation, cellular differentiation, and tissue‐specific functions [149]. For example, ethanol treatment of human ESC‐derived cortical neurons upregulated NMDA receptor subunit genes *GRIN1* (NR1) and *GRIN2B* (NR2B), indicating enhanced glutamatergic signaling and potential excitotoxic stress [150]. These changes mirror adaptations observed in animal models and human AUD, where the altered NMDA receptor function contributes to tolerance, dependence, and withdrawal. Building on this, genetic studies implicate downstream signaling molecules, such as FYN kinase, in alcohol dependence. Using iPSC‐derived neurons, researchers employed gene editing to introduce or correct AUD‐associated FYN variants, revealing changes in NMDA receptor phosphorylation, calcium dynamics, and neuronal excitability [151]. This suggests that the FYN 5^′^ UTR variant rs706895 may modulate vulnerability to alcohol dependence by altering the FYN expression. Overall, this iPSC‐derived neuronal model provides mechanistic insights into how genetic risk factors influence NMDA receptor signaling and neuronal function.

Alcohol exposure also alters DNA methylation and expression of *DPPA4*, a chromatin modifier essential for pluripotency, in both hESCs and placental tissues [139]. Downregulation of *DPPA4* during mesodermal and ectodermal differentiation suggests its involvement in disrupted early neurodevelopment. Along with genes such as *FOXP2* and *TACR3*, it could serve as a biomarker for PAE [139]. Chronic intermittent alcohol (CIE) exposure of iPSC‐derived cortical neurons disrupts regional patterning, synaptic function, axon guidance, and cell type specification. Notably, upregulation of WNT signaling drives a shift toward posterodorsal progenitor gene expression at the expense of anteroventral progenitors, paralleling behavioral and morphological phenotypes observed in animal models and patients with fetal alcohol syndrome [140]. These findings suggest that early alcohol exposure fundamentally alters cortical regionalization, potentially contributing to cognitive and behavioral deficits in fetal alcohol spectrum disorders (FASDs).

Moreover, iPSC‐derived microglial models highlight the interplay of genetic susceptibility and ethanol exposure. Recent work leveraging iPSC technology stratified donors by AUD polygenic risk score (PRS), calculated using genome‐wide significant variants from addiction genome‐wide association study (GWAS) meta‐analyses and weighted by effect size. Human microglia derived from iPSCs from individuals with AUD high‐PRS (diagnosed with AUD; *n* = 8) and low‐PRS (unaffected; *n* = 10) were directly compared. Microglia derived from high‐PRS individuals exhibited enhanced phagocytosis, increased CLEC7A expression, and morphological activation following ethanol exposure compared to controls [152]. In co‐culture with iPSC‐derived neurons, these high‐PRS microglia reduced synapse numbers after alcohol exposure, suggesting excessive synaptic pruning [152]. These findings provide compelling evidence that genetic risk factors, captured by PRS, shape neuroimmune responses and neuronal interactions in AUD, with potential implications for understanding individual vulnerability to addiction.

Additionally, chronic ethanol exposure in iPSC‐derived cardiomyocytes caused cell death, oxidative stress, abnormal calcium handling, impaired contractility, and disrupted structural development. Proteomic profiling revealed dysregulation of apoptosis, contractile proteins, extracellular matrix components, and signaling pathways including Wnt and TGF‐β [141]. By recapitulating these molecular and functional hallmarks of alcohol‐induced cardiomyopathy, this model provides a translational platform for dissecting ethanol‐induced cardiotoxicity mechanisms and screening cardioprotective interventions in individuals with AUD.

Together, ESC and iPSC models provide versatile, human‐relevant platforms to study the molecular, genetic, neuroimmune, developmental, and cardiotoxic effects of alcohol exposure. These studies identify candidate genes such as *DPPA4*, reveal cortical regionalization imbalances, demonstrate polygenic risk‐dependent microglial effects, and elucidate cardiomyocyte dysfunction. Overall, they advance understanding of AUD and FASDs and offer paths for biomarker discovery and therapeutic development.

### 5.3. ESC‐ and iPSC‐Derived Cellular Models for Studying Stimulant Use Disorder

Human ESC‐ and iPSC‐derived cellular models facilitate investigation of the cellular effects of stimulant exposure, including recreational drugs such as cocaine, amphetamines, and novel psychoactive substances (NPS). iPSC‐derived cardiomyocyte models show that these substances can directly alter cardiac function parameters, such as the beat rate, spike amplitude, and field potential duration, in a concentration‐dependent manner [142]. Amphetamines, MDMA (Ecstasy/Molly), and synthetic cathinones like MDPV affected cardiomyocyte function at concentrations relevant to human exposure, while cell viability remained largely unaffected except at much higher doses. These results suggest that stimulant‐induced tachycardia likely arises from indirect mechanisms, whereas prolonged QTc intervals may reflect direct cardiomyocyte effects. Beyond cardiotoxicity, hPSC‐derived neural models shed light on the neurodevelopmental consequences of stimulants.

Using a human neocortical differentiation protocol that recapitulates dorsal forebrain patterning and cortical layering, cocaine exposure during early neocorticogenesis accelerated neuronal differentiation and enhanced neurogenesis of multiple cortical neuronal subtypes [143]. These effects were diminished by cytochrome P450 inhibition, suggesting that metabolic processing mediates cocaine’s impact. Together, these studies demonstrate the utility of hPSC‐derived cardiomyocytes and cortical neurons for investigating stimulant use mechanisms, highlighting both cardiovascular and neurodevelopmental vulnerabilities associated with stimulant exposure.

In conclusion, human ESC‐ and iPSC‐derived cellular models offer versatile platforms for studying the molecular, cellular, and developmental effects of alcohol and drug addiction. In OUD, these models reveal how genetic variation, morphine exposure, and withdrawal alter dopaminergic neurons and midbrain progenitors. For AUD, iPSC studies highlight epigenetic regulators, microglial activity linked to polygenic risk, cortical patterning changes, and cardiomyocyte dysfunction. In stimulant use disorder, iPSC‐derived cardiomyocytes and cortical neurons demonstrate cardiotoxic and neurodevelopmental effects of drugs such as cocaine, amphetamines, and MDMA. Overall, stem cell‐based cellular models provide mechanistic insights and open avenues for biomarker discovery and therapeutic development.

## 6. Stem Cell‐Derived Organoids as Models for OUD, AUD, and Stimulant Use Disorder

Advances in organoid technology have yielded models that recapitulate aspects of human neural networks not captured by stem cell cultures. The diverse populations of neuronal and glial cells within organoids provide a unique opportunity to study how addictive substances impact neural circuits, synaptic connectivity, gene expression, and developmental pathways [107, 108, 153]. Importantly, organoid models offer insight into region‐specific vulnerabilities and network‐level responses to drugs of abuse, complementing findings from ESC‐ and iPSC‐derived cell cultures [97, 154]. In this subsection, we review recent applications of brain organoids in alcohol and drug addiction research (Table 4), highlighting their ability to capture the molecular, structural, and functional effects of addictive substances and their potential for therapeutic discovery.

**Table 4:** Stem cell‐derived organoids for alcohol and drug addiction research.

Type of organoid	Type of substances	Findings	References
Midbrain organoids	Opioid (fentanyl)	Chronic fentanyl disrupted neuronal subtype specification and synaptic activity; acute exposure increased dopamine release without altering developmental gene expression	Kim et al. [155]
Forebrain organoids	Opioid (oxycodone, buprenorphine)	Buprenorphine modulated transcription mainly in glial cells; oxycodone induced type I interferon signaling across neurons and glia	Ho et al. [139]
Cortical organoids	Opioid (methadone)	Chronic methadone altered genes for synaptogenesis, ECM organization, and ciliary function; TGFβ1 identified as upstream regulator	Dwivedi et al. [156]
Cortical organoids	Opioid (methadone, buprenorphine)	Methadone restricted organoid growth and neural activity via κ‐receptor and NMDA pathways; buprenorphine’s κ‐antagonism mitigated these effects	Yao et al. [157]
Cerebral organoids	Alcohol (prenatal/FASD)	Ethanol increased apoptosis, caused mitochondrial/cytoskeletal damage, and reduced oxygen consumption and ATP	Arzua et al. [158]
Brain organoids	Alcohol	Ethanol disrupted neurite outgrowth and neuronal maturation; altered GSX2, RSPO2, and Hippo pathway signaling	Zhu et al. [159]
Cortical organoids	Alcohol (prenatal)	Ethanol disrupted proliferation, increased apoptosis, impaired synaptogenesis, altered chromatin accessibility and histone modifications, and degraded network activity and Ca^2+^/cAMP signaling	Adams et al. [160]
Airway organoids	Stimulant (nicotine)	Nicotine injury mediated by nAChRs; N‐acetylcysteine (NAC) prevented damage by binding nicotine and reducing receptor interaction	Zheng et al. [161]
Cortical organoids	Stimulant (neonicotinoids/nicotine derivatives)	Neonicotinoids impaired neuron viability, disrupted synaptic proteins, activated microglia, and altered neurofilaments	Mariani et al. [162]
Cerebral organoids	Stimulant (nicotine/prenatal exposure)	Nicotine altered excitatory/inhibitory balance, reduced neural identity markers, and changed dopamine receptor expression; dysregulated neurogenesis and DNA binding genes	Proud et al. [163]
Cerebral organoids	Stimulant (cocaine)	Cocaine exposure disrupted gene expression and epigenetic profiles, impaired neural plasticity, and activated astrocyte mediated neuroinflammatory pathways	Davis et al. [164]
Ventral forebrain organoids	Stimulant (cocaine)	Identified immune and ROS‐dependent pathways affecting dopamine signaling; revealed cell‐type‐specific stimulant responses	Rudibaugh et al. [165]
Cerebral organoids	Stimulant (methamphetamine/prenatal exposure)	Methamphetamine induced widespread transcriptional changes in glia and progenitors, activated cytokines, complement, and inflammasome pathways	Dang et al. [166]

### 6.1. Organoid Models for Studying OUD

Organoid models have recently been used to investigate how opioids and pharmacotherapies for OUD affect human brain development and neural function. Kim et al. [155] developed an iPSC‐derived midbrain organoid system to assess the effects of acute and chronic fentanyl exposure during early development. Using single‐cell RNA sequencing (scRNA‐seq) of over 25,000 cells, they showed that chronic fentanyl treatment disrupted neuronal subtype specification and synaptic activity, while acute exposure increased dopamine release without significantly altering developmental gene expression [155]. This study represents the first single‐cell resolution analysis of opioid effects on human midbrain development, offering key insights into prenatal exposure consequences.

Building on this, another study used iPSC‐derived forebrain organoids from OUD patients to examine transcriptional responses to oxycodone and buprenorphine, the primary medications for OUD treatment [167]. Single‐nucleus RNA sequencing (snRNA‐seq) revealed distinct drug‐specific effects: buprenorphine primarily modulated transcription in glial cells, whereas oxycodone induced type I interferon signaling across multiple cell types, including neurons [167]. This work underscores the utility of patient‐derived organoids in identifying drug‐ and cell‐specific mechanisms relevant to the addiction and treatment. Similarly, Dwivedi et al. [156] demonstrated that chronic methadone exposure in human cortical organoids altered transcriptional programs involving synaptogenesis, extracellular matrix organization, and ciliary function, with TGFβ1 identified as a central upstream regulator. Yao et al. [157] compared methadone and buprenorphine effects, showing methadone restricted organoid growth and suppressed neural activity via κ‐opioid receptor signaling and NMDA antagonism, whereas buprenorphine’s κ‐antagonism mitigated these effects.

Beyond opioids, organoid platforms have been used for therapeutic discovery. Boutin et al. [168] developed a high‐throughput screening platform using iPSC‐derived cortical spheroids that display spontaneous, synchronous neural activity, enabling multiparametric profiling of drug responses across a 687‐compound neuroactive library. This study exemplifies how 3D models can bridge mechanistic insights and pharmacology in ways that are not achievable with traditional 2D cultures.

Together, these studies highlight the promise of brain organoids for modeling cellular and molecular opioid effects, medication‐based treatments, and candidate therapeutics. By capturing human‐specific developmental trajectories and network activity, organoids provide a powerful platform to probe drug mechanisms, assess treatment efficacy, and inform strategies to mitigate the neurological consequences of OUD.

### 6.2. Organoid Models for Studying AUD

Organoids have also been used to study AUDs, particularly focusing on PAE and underlying mechanisms of FASDs. These studies demonstrate how alcohol disrupts human‐specific neurodevelopmental processes that are difficult to replicate in animal models. For example, Arzua et al. [158] employed human iPSC‐derived cerebral organoids to model binge‐like ethanol exposure and found increased apoptosis, with neurons more vulnerable than astrocytes. In addition to cell death, ethanol‐treated organoids showed ultrastructural damage (such as mitochondrial disruption and cytoskeletal disorganization) and metabolic impairments, including reduced oxygen consumption and ATP production, suggesting compromised neuronal integrity during early development. Similarly, Zhu et al. [159] exposed iPSC‐based brain organoids to ethanol, observing disrupted neurite outgrowth and attenuated neuronal maturation. Transcriptomic analyses revealed altered expression of developmental regulators such as GSX2 and RSPO2 and perturbation of the Hippo signaling pathway, critical for cell proliferation and differentiation [159]. These molecular alterations indicate that alcohol interferes with neuronal lineage specification and structural maturation essential for circuit assembly.

Extending this work, Adams et al. [160] used cortical organoids to study the epigenetic and functional effects of PAE. Their analyses demonstrated impaired proliferation and cell cycle progression, increased apoptosis, and defective synaptogenesis [160]. At the epigenetic level, ethanol exposure led to significant changes in histone modifications and chromatin accessibility, underscoring profound alterations in developmental timing and gene regulation. Functional assays such as calcium imaging and multi‐electrode array recordings revealed impaired network activity and disrupted calcium and cAMP signaling, illustrating how alcohol compromises both cellular differentiation and emergent electrophysiological properties of developing neural networks.

Collectively, these studies reveal that ethanol produces dose‐ and stage‐dependent effects on cell survival, differentiation, metabolism, and synaptic maturation, with neurons particularly susceptible. Organoid models provide a unique human platform to integrate transcriptomic, epigenetic, and functional data, offering novel mechanistic insights into AUD and FASD origins. Although challenges remain, such as limited organoid maturation and the lack of vascular and immune components [169], this work lays a strong foundation for future organoid applications to model alcohol‐related neurodevelopmental disorders and test therapeutic interventions.

### 6.3. Organoid Models for Studying Stimulant Use Disorder

Human brain and airway organoids have emerged as powerful tools to model the effects of nicotine and stimulants on neurodevelopment and tissue function, providing insights into molecular, cellular, and circuit‐level mechanisms difficult to study in animal models. For instance, Zheng et al. [161] developed patient tissue‐derived airway organoids to study smoking‐induced injury and evaluate therapeutic effects of N‐acetylcysteine (NAC). These organoids, comprising ciliated, basal, goblet, and myofibroblast‐like cells, feature apical‐out and basal‐in polarity with beating cilia responsive to nicotine exposure [161]. Their study revealed that nicotine‐induced injury is mediated by nicotinic acetylcholine receptors (nAChRs) and that NAC confers protection not only via antioxidation but also by binding nicotine and reducing its interaction with nAChRs, highlighting organoids’ utility for dissecting substance‐specific mechanisms and therapeutic responses.

Human iPSC‐derived cortical organoids have also been utilized to investigate neurodevelopmental toxicity from nicotine and related compounds. Mariani et al. [162] found that neonicotinoids (nicotine‐derived insecticides) impair neuron viability, disrupt synaptic protein expression and neurofilament structures, and activate microglia in both human cortical organoids and prenatal mouse models. Proud et al. [163] modeled prenatal nicotine exposure using cerebral organoids and observed lasting alterations in cortical excitatory/inhibitory balance, reduced neural identity markers, and altered dopamine receptor expression. Transcriptomic analyses revealed dysregulation of genes related to neurogenesis, embryonic development, and DNA binding, indicating that nicotine‐induced effects persist across development and may underlie susceptibility to later mood and anxiety disorders.

Organoids have also been instrumental in elucidating the effects of stimulants like cocaine and methamphetamine on human neurodevelopment. Davis et al. [164] applied scRNA‐seq and ATAC sequencing to iPSC‐derived cerebral organoids exposed to cocaine, revealing disrupted gene expression and epigenetic landscapes, impaired neural plasticity, and activation of astrocyte‐mediated neuroinflammatory pathways—mechanisms that may contribute to cognitive and emotional dysfunction. Similarly, Rudibaugh et al. [165] used ventral forebrain organoids to study dopamine signaling in response to cocaine, identifying robust immune responses and reactive oxygen species‐dependent pathways, highlighting the complex, cell‐type‐specific effects of stimulants on developing neural circuits. Dang et al. [166] investigated prenatal methamphetamine exposure with cerebral organoids and single‐cell transcriptomics, uncovering widespread transcriptional changes in astrocytes, oligodendrocytes, and neural progenitors. Methamphetamine exposure triggered neuroinflammatory responses including cytokine and inflammasome activation, upregulation of immediate early genes and complement factors, and increased apoptosis, revealing how stimulants disrupt neurodevelopment via coordinated neuroimmune and cellular signaling pathways.

Collectively, these studies demonstrate the versatility and translational relevance of human organoids in addiction research (Table 4). Alcohol and drug exposures induce persistent changes in neuronal differentiation, synaptic organization, glial function, and gene regulation, revealing both the molecular and functional consequences of substance use. Organoid models enable detailed investigation of neuroinflammatory pathways and epigenetic remodeling and offer a human‐relevant platform to test potential therapeutics. By overcoming species‐specific limitations of animal models and allowing detailed examination of the developmental stage and cell‐type vulnerabilities, organoids have become essential tools for understanding the complex effects of addictive substances and for guiding prevention and treatment strategies.

## 7. Stem Cell‐Derived Organoids‐on‐Chips: Innovative Platforms for Addiction Research

Building upon conventional organoids, organoid‐on‐a‐chip platforms integrate microfluidic engineering with 3D neural cultures to enhance nutrient delivery, introduce vascular‐like features, and provide greater experimental control and reproducibility [170]. These systems address several limitations of standard organoids, including heterogeneity and limited maturation [118] and offer novel opportunities to investigate long‐term drug exposure, dynamic responses to addictive substances, and patient‐specific variability [127]. In this subsection, we highlight emerging studies leveraging organoid‐on‐a‐chip technology in alcohol and drug addiction research (Table 5), emphasizing how these platforms advance experimental precision, reproducibility, and translational potential for drug screening and therapeutic testing.

**Table 5:** Organoids‐on‐chips for alcohol and drug addiction research.

Model type	Origin of tissues/cell type	Type of substances	Findings	References
Multi‐organ microphysiological system (MPS)	Brainstem respiratory centers, liver, cardiac, skeletal muscle tissues	Opioid (methadone, overdose model)	Modeled acute opioid overdose and recovery; methadone reduced neural activity consistent with overdose; naloxone restored function	Patel et al. [171]
Spinal microphysiological system (MPS)	Human spinal cord organoid with 3D‐printed holder	Opioid (tolerance)	Chronic opioid exposure led to receptor adaptations, altered synaptic strength, and network excitability	Cai et al. [172]
Multi‐organ MPS (Liver, Heart, Lung)	Human tissue constructs	Alcohol (systemic effects)	Alcohol exposure reduced viability, urea, albumin secretion, and increased cell damage markers; modeled multi‐organ effects of alcohol.	Skardal et al. [173]
Alcoholic Liver Disease (ALD) Liver‐Chip	Primary human hepatocytes and stellate cells	Alcohol (chronic exposure/ALD)	Ethanol exposure caused lipid accumulation, oxidative stress, fibrosis, and bile canalicular remodeling	Nawroth et al. [174]; Liu et al. [141]
Brain organoid‐on‐chip	Human iPSC‐derived brain organoids	Stimulant (nicotine/prenatal exposure)	Nicotine disrupted cortical layer formation, reduced synaptic protein expression, and impaired neural progenitor proliferation; mirrored human prenatal nicotine exposure outcomes	Wang et al. [175]
Vascularized liver‐on‐chip	Liver organoids integrated with perfusable vascular channels	Stimulant (nicotine)	Chronic nicotine disrupted vascular barrier function, hepatocyte metabolism, and induced inflammatory signaling; demonstrated systemic nicotine toxicity	Wang et al. [176]

### 7.1. Technical Challenges and Considerations in Organoid‐on‐Chip Development

Despite their promise, organoid‐on‐chip platforms face several critical technical hurdles that must be addressed to enable broader adoption in addiction research. Chip fabrication and standardization remain primary challenges; most platforms are custom‐designed, limiting reproducibility and cross‐laboratory comparisons. Scaling to a clinically relevant throughput is another bottleneck. Current systems typically accommodate 1–10 tissue units per chip [177, 178], making large‐scale drug screening or patient‐derived personalized testing economically unfeasible without significant engineering innovation.

Multi‐organ integration adds complexity. Coordinating tissue maturation, maintaining distinct microenvironments, and achieving appropriate cell‐type ratios across multiple organ modules require sophisticated fluid dynamics modeling and careful tuning of culture conditions [179, 180]. Hypoxia remains problematic in densely packed organoids despite improved nutrient delivery; achieving oxygen gradients that mirror in vivo physiology is technically demanding [181]. Long‐term culture stability is also critical. Maintaining viable, functional multi‐tissue systems for weeks to months without contamination or progressive dedifferentiation demands robust perfusion strategies and dynamic medium composition control. Additionally, standardized readouts and quantification methods remain underdeveloped relative to organoid advancement. Most current systems rely on endpoint measurements (immunostaining and gene expression), limiting insight into dynamic processes. Integration of real‐time biosensors (electrophysiology, oxygen sensors, and metabolic sensors) requires biocompatible materials and seamless interfacing with tissue, a field still in its infancy [182]. Addressing these challenges is essential for transitioning the organoid‐on‐chip technology from proof‐of‐concept demonstrations to reproducible, scalable tools for addiction research.

### 7.2. Organoid‐on‐a‐Chip Models for Studying OUD

Organ‐on‐a‐chip models have emerged as innovative systems to study OUD, providing relevant human models that capture both the cellular and systemic effects of opioid exposure. Human brain organoids‐on‐chips integrated with sensors enable continuous monitoring of neuronal activity, synaptic connectivity, and network dynamics, yielding insights into how opioids disrupt neural function and induce neurotoxicity [183]. These systems facilitate precise temporal and dose‐dependent analyses of both acute and chronic opioid effects, making them invaluable for evaluating potential treatments in a controlled, physiologically relevant neural context. Beyond the brain, organoids‐on‐chips incorporating brainstem respiratory centers, liver, heart, and skeletal muscle tissues have been developed to model acute opioid overdose physiology and recovery [171]. Using these platforms, methadone exposure reduced neural activity in a manner consistent with opioid overdose, whereas naloxone treatment effectively restored neural function. Simultaneous monitoring of cardiac and other organ‐specific modules revealed unintended cardiac toxicities, suggesting potential off‐target pharmacological effects [171].

Complementing these models, human spinal cord organoids‐on‐chips have provided mechanistic insights into opioid‐induced tolerance [172]. Each spinal organoid‐on‐a‐chip consisted of a flattened human spinal cord organoid mounted on a 3D‐printed holder, which reduced hypoxia and necrosis, thus promoting neuronal maturation, activity, and development. Cai et al. [172] demonstrated that prolonged opioid exposure induces neurochemical changes associated with tolerance, including receptor‐level adaptations, modified synaptic strength, and altered network excitability, thereby mirroring the clinically observed decline in analgesic efficacy.

Collectively, these studies underscore that organoid‐on‐a‐chip systems can model multiple facets of OUD, including neurotoxicity, network disruption, overdose physiology, and tolerance mechanisms. These versatile platforms offer translationally relevant tools for mechanistic studies, therapeutic testing, and drug development, overcoming limitations inherent in conventional 2D cultures or animal models.

### 7.3. Organoid‐on‐a‐Chip Models for Studying AUD

Organoid‐on‐a‐chip models have also become valuable tools for investigating AUD, enabling the examination of alcohol’s impact within a 3D architecture. Early liver‐on‐a‐chip models replicated the human liver’s structure and function, enabling studies of alcohol‐induced alterations in drug metabolism and liver injury [174, 184]. Building on this, Skardal et al. [173] developed a multi‐organ microphysiological system integrating liver, heart, and lung tissues on a single platform, facilitating the study of alcohol’s systemic effects on hepatic metabolism, cardiac function, and pulmonary responses. Upon exposure to ethanol, cell viability and secretion of urea and albumin decreased, while markers of cellular damage increased [173, 174]. This advanced platform therefore provides a comprehensive model for understanding the systemic pathology of AUD.

A recent advancement is the alcoholic liver disease (ALD) liver chip, which integrates primary human hepatocytes and stellate cells within a microfluidic device. This system replicates chronic alcohol exposure and models the progression of alcohol‐induced liver injuries, including fibrosis and cirrhosis. Nawroth et al. [174] exposed the Liver‐Chip to ethanol for 48 h and observed lipid accumulation, increased oxidative stress, gene expression changes, and bile canalicular remodeling. These findings demonstrate the Liver‐Chip’s utility in studying ALD pathophysiology and evaluating potential therapeutics. Despite these advances in liver pathology modeling, further research is critical to elucidate the complex neurobiological mechanisms underlying AUD.

### 7.4. Organoid‐on‐a‐Chip Models for Studying Stimulant Use Disorder

Organoid‐on‐a‐chip models investigating stimulant use disorders have primarily focused on nicotine, due to its high prevalence and well‐documented health risks. Recent studies have utilized organoid‐on‐a‐chip platforms to explore nicotine’s effects on neural development, hepatic function, and respiratory tissues, as well as to identify potential interventions. Wang et al. [175] developed human brain organoids‐on‐chips to model prenatal nicotine exposure, allowing precise dosing during key neurodevelopmental stages. Nicotine disrupted normal cortical layer formation, reduced synaptic protein expression, and impaired the proliferation of neural progenitors. These results parallel clinical observations linking maternal smoking with impaired neurodevelopment and heightened neuropsychiatric risk in offspring, highlighting organoid‐on‐a‐chip systems’ value in studying stimulant‐induced developmental neurotoxicity.

Expanding upon this, Wang et al. [176] employed a vascularized liver‐on‐chip model to examine nicotine‐induced hepatic dysfunction. The system incorporated perfusable vascular channels with liver organoids, enabling dynamic interactions among hepatocytes, endothelial cells, and circulating factors [176]. Chronic nicotine exposure impaired vascular barrier integrity, disrupted hepatocyte metabolism, and activated inflammatory signaling. These findings emphasize nicotine’s toxicity beyond the nervous system, affecting peripheral organs and underlining the importance of multi‐organ models. The integrated vascular channels also allowed investigations into nicotine’s effects on tissue perfusion and endothelial stability.

Together, these studies demonstrate that organoid‐on‐a‐chip models capture nicotine toxicity’s complex effects across the neural, hepatic, and respiratory systems. This body of work highlights the promise of organoid‐on‐a‐chip technology to propel stimulant use disorder research forward. These platforms hold the potential to expand research beyond nicotine to other stimulants, enabling systematic exploration of organ‐specific vulnerabilities, drug interactions, and personalized therapeutic strategies.

## 8. Advancing Addiction Pharmacology Through Stem Cell‐Based Approaches

In addition to disease modeling, stem cell‐derived systems are emerging as valuable tools for pharmacological development. These platforms offer unique opportunities for drug screening and efficacy testing, the identification of novel therapeutic targets, and the advancement of personalized treatment strategies.

### 8.1. Drug Screening and Efficacy Testing

Stem cell‐derived systems more accurately replicate human neural, hepatic, and systemic environments compared to traditional animal models or immortalized cell lines. For example, Kong et al. [185] utilized a skin stem cell‐based gene delivery platform for treating AUD or polysubstance abuse. They demonstrated that mesenchymal stem cell (MSC)‐derived exosomes improved memory, enhanced hippocampal neurogenesis, and reduced neuroinflammation in a mouse model of methamphetamine addiction [185]. Another study reported that intranasal administration of the secretome from activated adipose‐derived MSCs reduced chronic intake and relapse behaviors for nicotine and alcohol in rodents [186]. This treatment also decreased oxidative stress and neuroinflammation while restoring glutamate homeostasis via the upregulation of glutamate transporter 1 (GLT1). These findings highlight how stem cell‐derived therapies can be evaluated for both efficacy and safety in preclinical addiction models.

### 8.2. Identification of New Therapeutic Targets

Stem cell‐derived models facilitate the discovery of novel therapeutic targets by elucidating the molecular mechanisms underlying alcohol and drug addiction. Fallahi et al. [187] showed that MSC‐derived exosomes enhance neurogenesis and cognitive function in a mouse model of methamphetamine addiction. The treatment promoted hippocampal neurogenesis and memory performance, while suppressing microglial activation and inflammatory signaling pathways [187]. These findings suggest that pathways modulated by MSC exosomes, such as neurogenesis and anti‐inflammatory signaling, represent promising targets for stimulant use disorder treatment. This study exemplifies how stem cell‐based approaches can uncover actionable cellular and molecular pathways for pharmacological intervention in alcohol and drug addiction.

### 8.3. Personalized Treatment

Stem cell‐derived systems are crucial for advancing personalized medicine. iPSCs generated from patients retain their unique genetic and epigenetic signatures that influence drug response. These patient‐specific models enable researchers to assess individual variability in drug efficacy and toxicity [188]. Furthermore, the iPSC technology allows differentiation into disease‐relevant neural subtypes, facilitating precise modeling of disease pathways and drug responses. Using such models, researchers can predict therapeutic efficacy, anticipate adverse effects, and optimize treatment strategies for individual patients.

Beyond in vitro studies, direct therapeutic applications of iPSCs are under investigation. For example, Kirkeby et al. [189] transplanted iPSC‐ and ESC‐derived dopaminergic neurons into preclinical Parkinson’s disease models to evaluate safety, tumorigenicity, biodistribution, and functional recovery. These cells were produced under Good Manufacturing Practice (GMP) conditions, ensuring reproducible quality and efficacy across batches, and are currently being tested in an early‐phase human clinical trial for patients with moderate Parkinson’s disease.

## 9. Conclusions and Perspectives

Human ESCs and iPSCs enable patient‐specific neural modeling, while organoids and organoid‐on‐a‐chip systems recapitulate complex tissue interactions. Together, these platforms overcome traditional model limitations and provide human‐relevant platforms for understanding cellular, molecular, and network‐level mechanisms in addiction.

These models are utilized in mechanistic studies that can reveal how addictive substances alter neuronal function, signaling pathways, and network connectivity. The versatility of stem cell and organoid technology allows researchers to model multiple aspects of alcohol and drug addiction within the same experimental framework. Integrating patient‐derived cells with complex organoid models enhances understanding of individual differences in addiction susceptibility and treatment response while also offering a platform to investigate potential therapeutic strategies.

To date, the number of stem cell studies on alcohol and drug addiction remains limited. Nonetheless, these innovative stem cell‐derived systems hold great promise for advancing research in this field by offering mechanistic insights and experimental platforms that can accelerate the development of effective, individualized strategies for understanding and treating alcohol and drug addiction.

**Nomenclature**
2D: Two‐dimensional
3D: Three‐dimensional
ALD: Alcoholic liver disease
AUD: Alcohol use disorder
ChAT: Choline acetyltransferase
CIE: Chronic intermittent alcohol exposure
CUD: Cocaine use disorder
CYP: Cytochrome P450
DAT: Dopamine transporter
ESC: Embryonic stem cell
FASD: Fetal alcohol spectrum disorders
GLT1: Glutamate transporter 1
GMP: Good manufacturing practice
hPSC: Human pluripotent stem cell
MSC: Mesenchymal stem cell
NAC: N‐acetylcysteine
nAChRs: Nicotinic acetylcholine receptors
NPS: Novel psychoactive substances
OUD: Opioid use disorder
PAE: Prenatal alcohol exposure
PMI: Postmortem interval
PRS: Polygenic risk scores
PSD‐95: Postsynaptic density protein 95
SA: Self‐administration
scRNA‐seq: Single‐cell RNA sequencing
snRNA‐seq: Single‐nucleus RNA sequencing
SUD: Substance use disorder
SV2: Synaptic vesicle protein 2
SYN: Synaptophysin
TH: Tyrosine hydroxylase
VGLUT: Vesicular glutamate transporter
VNTR: Variable number tandem repeat polymorphism.

## Author Contributions

Ji Sun Koo drafted the review. Huiping Zhang revised the manuscript and finalized it for submission.

## Funding

This work was supported by the National Institute on Alcohol Abuse and Alcoholism (Grant R01 AA029758) and the National Institute of Child Health and Human Development (Grant R01 HD113143).

## Conflicts of Interest

The authors declare no conflicts of interest.

## Data Availability

Data sharing is not applicable to this article, as no new datasets were generated or analyzed during this study.
